# Awareness and Attitude of First Aid Seizures Management among Medical Undergraduate Students, Tobruk University, Libya

**DOI:** 10.1055/s-0045-1809427

**Published:** 2025-06-12

**Authors:** Zinelabedin Mohamed, Aisha A.J. Marajie, Nahla B.S. Najem, Nooralhuda G.M. Elmarimy, Heba M.A. Abdulmoula, Mohammad Amin Aly El-Din

**Affiliations:** 1 Department of Internal Medicine, Faculty of Medicine, Tobruk University, Tobruk, Libya; 2 Clinical Research Department, Abou Al Monagga Central Hospital, Health Affairs Directorate, Ministry of Health and Population, Qalyubia, Egypt

**Keywords:** awareness, first Aid, Libya, medical students, seizure management, Tobruk University

## Abstract

**Background:**

Adequate knowledge of first aid for seizures is crucial for medical students, who will eventually be responsible for managing epilepsy patients. The aim of the study was to assess the awareness and attitudes of medical undergraduate students at Tobruk University regarding first aid seizure management.

**Methods:**

A cross-sectional study was initiated in July 2023 using an online questionnaire that was prevalidated. The questionnaire gathered data on sociodemographic features, a knowledge of seizures and epilepsy, first aid practices, and attitudes toward epilepsy among 317 medical undergraduate students.

**Results:**

While 72.9% of students correctly identified a seizure, there were different beliefs about causes, such as some that attributed seizures to supernatural causes (14.2%). There were also deficiencies seen in the knowledge of epilepsy management that included antiepileptic drug treatment duration. It was alarming that 41.6% of students thought that the insertion of some objects into the mouth of a person having a seizure was first aid, which is a well-known hazardous approach. Only 23.6% were correct in the answers that involved the placement of the person in a semiprone position to prevent choking.

**Conclusion:**

The study at Tobruk University revealed significant knowledge gaps among medical students about seizure management, with 72.9% correctly identifying a seizure's basic definition, but 41.6% incorrectly believing that inserting objects into a seizing person's mouth is helpful, and only 23.6% knowing the correct first aid position. Students also demonstrated misconceptions about epilepsy causes, including supernatural beliefs, highlighting an urgent need for targeted educational interventions to improve understanding and prepare future health care professionals.

## Introduction

Epilepsy is a chronic neurological disorder characterized by recurrent, unpredictable seizures that significantly impact patients' quality of life. 1 Medical students, as future health care practitioners, play a crucial role in understanding and managing epilepsy, yet studies have consistently revealed knowledge gaps and persistent misconceptions about seizure management. 2 3 The prevalence of epilepsy varies globally, with substantial regional differences in awareness and attitudes toward the condition. 4 In many developing countries, cultural beliefs and limited medical education contribute to misunderstandings about seizures, often leading to inappropriate first aid interventions and social stigmatization. 5 These misconceptions can have serious consequences, potentially exacerbating patient risks during seizure episodes. Medical education represents a critical opportunity to address these knowledge deficits and reshape attitudes toward epilepsy. Research has demonstrated that targeted educational interventions can effectively improve health care professionals' understanding of seizure management and reduce associated stigma. 6 However, the effectiveness of such interventions depends on comprehensive assessment of existing knowledge and misconceptions. In the Libyan context, limited research has explored medical students' awareness and attitudes toward seizure management. Understanding the current knowledge landscape is essential for developing tailored educational strategies that can improve future health care practitioners' competence in managing epilepsy. 7 This study aims to bridge this knowledge gap by comprehensively examining medical undergraduate students' awareness, attitudes, and first aid practices related to seizures at Tobruk University. By investigating students' understanding of seizure definitions, causes, types, and appropriate first aid measures, we seek to identify critical areas for curriculum enhancement. The findings will provide insights into the current state of epilepsy education and guide the development of targeted interventions to prepare medical students for effective patient care.

## Materials and Methods

### Study Design

This research was conducted during July 2023, so, as mentioned earlier, it is a cross-sectional study, which means the participants were not timed but were just surveyed at one instant involving participants of Tobruk University's medical school taking online questionnaire, which is considered one of the best ways to access those students in this unique study. Tobruk knowledge of, and attitudes toward, first aid seizure management were assessed among undergraduate medical students of Libya. Treatment of part in this study questionnaire was taken from previously validated study, and thus it consists of questions that were used for both part one and part two in the previous study. 8 The questionnaire was distributed among medical students in their clinical and preclinical years as well as interns of Faculty of Medicine at Tobruk University.

### Instrument

The online questionnaire was prevalidated and designed to investigate participants' knowledge, attitudes, and behaviors regarding seizures and first aid practices. 8 The questionnaire was made to fit the context of this study and the fact that it was translated and clarified to the learners makes it the one made use of in this research study. The questions in the questionnaire could be categorized into four sections: (1) Sociodemographic information: This section gathered information such as the participant's age, sex, marital status, year of study in the medical program, and place of domicile (urban/rural). (2) Knowledge about seizures and epilepsy: This part has questions that are in multiple-choice format and gave students the indication of their knowledge about types of seizures; participants were asked to select one option that they believed represented the most common or representative type of seizure, rather than identifying all correct seizure types. (3) First aid for seizures: The scenario-based questions in this section were used to find out if students know the correct first aid measures for seizures including do's and don'ts and any false beliefs about seizures such as these ones. (4) This part used Likert-scale items to measure the factor of attitudes toward epilepsy: several students' views on the social and personal consequences of this condition as well as their perceived level of confidence in giving first aid for seizures were considered. The questionnaire was conducted using Google Forms, an online data gathering and dissemination platform. The link to the questionnaire was sent out to potential participants through various channels, such as student email lists and social media groups. To ensure anonymity and confidentiality, no personal identifying information was collected, and data were securely saved on the Google Forms platform.

### Sample Size Estimation

The sample size was estimated utilizing the Epitools sample size calculator. The sample size was determined by means of the single population proportion formula, with the assumption that 50% of students know the first aid measures for epilepsy. The parameters set for this computation were the 95% confidence level and a 5% margin of error. The sample size was initially found to be 292. In consideration of a 5% nonresponse rate, the final sample size was adjusted pragmatically to 307 participants. *Statistical analysis* : Data entry and analysis were carried out using SPSS for Windows, version 27.0. Data analysis included the use of both descriptive and inferential statistics. The mean and standard deviation were calculated for normally distributed data, whereas for nonnormally distributed data, the median and quartile range were used. The chi-square test of independence was applied to analyze the data with a significance level of *p* -value set at less than 0.05.

### Ethical Considerations

Ethical approval for this study was obtained from the Research Ethics Committee of the University of Tobruk, with a reference number NBC:009. H.23.4. All participants were informed about the purpose of the study and provided their consent before participating. The confidentiality and anonymity of the participants were maintained throughout the research process.

## Results

### Demographic Characteristics

A total of 317 medical undergraduate students participated in the study. Most of the participants were female (79.8%), single (94.3%), and resided in urban areas (92.7%). The median age of the participants was 22 years (1st–3rd quartile: 20–24). Students from all 6 years of the medical program participated, with the highest representation from the 4th year (30.9%), followed by the 1st year (21.8%) and 2nd year (22.4%).

### Knowledge of Seizures and Epilepsy

- Definition of a seizure: Most students ( *n* = 231, 72.9%) correctly identified a seizure as an abnormal electrical discharge in the brain. However, misconceptions were also prevalent, with some students associating seizures with abnormal movements (12.9%), demonic possession (8.8%), or divine punishment (5.4%).
- Causes of epilepsy: The most identified causes of epilepsy were head injury (28.7%), genetic disease (25.2%), and brain tumor (17.0%). Notably, a proportion of students held beliefs in supernatural causes, such as an evil spirit (6.3%) or divine punishment (3.8%).
- Types of seizures: When asked to identify types of seizures, *n* = 110, 34.7%, of students selected tonic-clonic seizures (characterized by rigidity followed by jerking), which are the most clinically recognizable seizure type. Other responses included complex partial seizures (24.9%), simple partial seizures (19.9%), absence seizures (8.2%), and atonic seizures (12.3%).
- Curability of epilepsy: Uncertainty regarding the curability of epilepsy was evident, with 46.7% of students believing it “may be” curable. While 34.4% recognized that epilepsy can be cured, a significant proportion (18.9%) believed it to be incurable.
- Duration of antiepileptic drug (AED) treatment: A slight majority of students (51.1%) correctly understood that AEDs often need to be taken for life. However, a substantial proportion held incorrect beliefs, such as taking them for 2 to 5 years (27.1%), 3 to 6 months (7.3%), only during an episode (9.5%), or only on the full moon (5.0%).

### Attitudes and Practices Related to Epilepsy

- Consequences of epilepsy: The most widely perceived consequence of epilepsy was the restriction from driving a motor vehicle (48.2%). Other misconceptions included the inability to marry (3.5%), get pregnant (4.4%), breastfeed (2.8%), or engage in sexual intercourse (6.6%).
- First aid for seizures: A concerning finding was the high proportion of students (41.6%) who believed that placing something in the mouth of a seizing person to prevent tongue biting was appropriate. This practice is harmful and can obstruct the airway. Only 23.6% correctly identified placing the person in a semiprone position to prevent choking as an appropriate first aid step.

### Other Findings

- Smoking habits: Most students (94.0%) reported being nonsmokers.
- Academic performance: The distribution of students' cumulative academic scores represented as the majority falling into the “Good” (41.3%) and “Very Good” (36.0%) categories. Awareness and attitude responses are presented in Table 1 .

**Table 1:** Awareness and attitude responses

		Count	Percentage (%)
What do you think a seizure is?	An abnormal electrical discharge in the brain	231	72.9
	An abnormal movement	41	12.9
	Demonic possession	28	8.8
	Divine punishment	17	5.4
What do you think causes epilepsy?	A head injury	91	28.7
	Alcohol withdrawal or heavy drinking	4	1.3
	An evil spirit	20	6.3
	Brain tumor	54	17.0
	Divine punishment for reneging on a vow	12	3.8
	Eating pork	4	1.3
	Genetic disease	80	25.2
	High fever	28	8.8
	Sleep deprivation	7	2.2
	Stroke	17	5.4
What are the types of seizures?	Loss of muscle strength and tone: the person collapses (atonic seizure)	39	12.3
	Lost awareness and physically disabled, repetitive involuntary movements (complex partial seizure)	79	24.9
	Rigid then jerking (tonic-clonic seizure)	110	34.7
	Staring spell, suddenly absent, loss of awareness (absence seizure)	26	8.2
	Unusual sensation or abnormal jerking with preserved awareness (simple partial seizure)	63	19.9
Do you think epilepsy can be cured?	May be	148	46.7
	No	60	18.9
	Yes	109	34.4
How long should antiepileptic drugs be taken?	2–5 years	86	27.1
	For 3–6 months	23	7.3
	For life	162	51.1
	Only during an episode	30	9.5
	Only on the full moon	16	5.0
What are the consequences of epilepsy?	Abruptly stop antiepileptic drugs during pregnancy	29	9.1
	Cannot get married	11	3.5
	Cannot get pregnant	14	4.4
	Must quit work	18	5.7
	No sexual intercourse	21	6.6
	Not able to lactate	9	2.8
	Should not allowed to drive a motor vehicle	153	48.2
	Should not drink alcohol beverages	19	6.0
	Should not eat pork	6	1.9
	Should not work with machinery	37	11.7
What should be done during a seizure?	Give an antiepileptic drug during the episode	49	15.5
	Place something in the mouth to prevent biting the tongue	132	41.6
	Place the person in a semiprone position to prevent choking	75	23.6
	Prevent injury during the episode	38	12.0
	Restrain the person and perform chest compressions (CPR)	23	7.3
Smoking	No	298	94.0
	Yes	19	6.0
Cumulative academic score	Excellent	55	17.4
	Good	131	41.3
	Poor/pass	17	5.4
	Very good	114	36.0

Abbreviation: CPR, cardiopulmonary resuscitation.

## Discussion

Note that 72.9% of students correctly identified that a seizure is an unusual discharge of electrical energy in the brain and the remainder had a misconception, for instance, associating seizures with abnormal movements (12.9%). It was lesser than high school students' (91.8%) study in Thailand. 8 The students in this study who believed that epilepsy is a hereditary disease (25.2%) were lower than the ones in Kerala (34%), Canada (45%), and Malaysia (67%). 9 10 11 But others believed it was due to demonic reason (8.8%) or divine punishment (5.4%). Similarly, Ismail et al reported that Muslims living in the United Kingdom believe that epilepsy is demonic. 12 These misconceptions align with the findings of similar studies and suggest that the cultural and educational backgrounds of the medical students influence the students' understanding of epilepsy and consequently this can be a cause of large treatment gaps as faith healers will be sought out by patients instead of qualified personnel. 13 14

Regarding the causes of epilepsy, the most commonly identified were head injury (28.7%), genetic disease (25.2%), and brain tumor (17.0%). Yet, it is worrying that most students believed that dignity is the main cause of epilepsy, blaming evil spirits (6.3%) or the fact that they had broken a promise that they had made to God (3.8%). For example, there was a similar tendency for supernatural associations such as “evil spirits” and “divine punishment for being unfaithful to God” to be seen as contributing factors (6.3 and 3.8%, respectively) as in other studies. 15 This shows that old beliefs still persist among medical students in our country, and these beliefs can influence the practice of medicine, including how they care for patients. 16

Medical students recognized the various types of seizures badly, with less than 50% of the students (34.4%) identifying tonic-clonic seizures correctly, followed by 24.9% who have believed them to be complex partial seizures and 8.2% who thought that absence seizures were the least common. A study that was conducted in Thailand indicates that, in contrast, the percentage of those at a high level of complexity was as follows: 97.2% of people had had at least one tonic-clonic seizure; 11.8% were complex partial seizures; and 33.6% had absence epilepsy. 8 This lack of knowledge can cause misdiagnosis and unsuitable treatment of epilepsy patients.

Regarding the management of epilepsy, 34.4% of students thought it could be treated, a number that is lower than the 46.3% of respondents who held that view in Ab Rahman's study. 11 However, most students, 46.7%, believed that epilepsy could be cured. Based on the data presented, the proportion of students with misperceptions regarding the prognosis of epilepsy was 18.9%. Some respondents mentioned inaccurate knowledge regarding the consequences of epilepsy, such as the discontinuation of AEDs during pregnancy (by 9.1%), the assumption that mothers are not able to breastfeed (2.8%), and the avoidance of sexual intercourse (6.6%). Most respondents (47.9%) realized that drivers with epilepsy should not have their permits. In addition, 11.7% of the respondents were aware that people with epilepsy want to avoid machines. This finding reinforces the notion that all aspects of the disease and its management are included in education and reflect the demand for fair and concise guidelines in medical curricula.

A very important finding was that a significant number of students (41.6%) thought that it was appropriate to take something to a seizing person's mouth to prevent biting the tongue. Instead of being helpful, this method can be damaging to the person with the seizure, who may asphyxiate, thus it is not recommended. Only 23.6% were successful in identifying putting the person in a semiprone position to allow airways to be open as correct first aid. This study's findings match studies by Kankirawatana, Dantas et al, and Fong and Hung. 17 18 19 As per the research done by Senanayake and Abeykoon, 64% of respondents, who had prior first aid experience with seizures, revealed hazardous practices like sticking wood in the person's mouth. 15 This highlights a critical requirement for complete reeducation and better training of medical students in first aid for seizures. 20

Notably, 94.0% of the students claimed to be nonsmokers, which may indicate a health- conscious population. Also, the distribution of cumulative academic scores showed that students at the highest level of achievement formed the bulk of the participants, with “Good” (41.3%) and “Very Good” (36.0%) being the two largest categories. This confirms that the information gap is not due to the lack of academic ability but mainly due to inadequate education on epilepsy and seizure management.

## Limitations and Future Directions

This study was limited to a single university in Libya and may not be generalizable to other contexts. Future research should explore these issues across multiple institutions and incorporate qualitative methods to gain deeper insights into students' beliefs and attitudes.

## Implications for Education and Practice

The findings underscore the crucial need to strengthen first aid seizure management education within medical curricula. Integrating interactive teaching methods, such as simulated seizure scenarios and case-based learning, could enhance knowledge retention and promote the adoption of evidence-based practices. 8 Moreover, incorporating epilepsy awareness campaigns within universities and the wider community can help dispel myths and reduce stigma. 10

## Conclusion

The study at Tobruk University revealed significant knowledge gaps among medical students about seizure management, with 72.9% correctly identifying a seizure's basic definition, but 41.6% incorrectly believing that inserting objects into a seizing person's mouth is helpful, and only 23.6% knowing the correct first aid position. Students also demonstrated misconceptions about epilepsy causes, including supernatural beliefs, highlighting an urgent need for targeted educational interventions to improve understanding and prepare future health care professionals.
